# The role of ultrasound in the diagnosis of the coexistence of primary hyperparathyroidism and non-medullary thyroid carcinoma

**DOI:** 10.1186/s12880-019-0306-8

**Published:** 2019-01-18

**Authors:** Jian Shen, Qiong Wu, Yan Wang

**Affiliations:** Department of Ultrasound in Medicine, Shanghai Jiao Tong University Affiliated Sixth People’s Hospital, Shanghai Institute of Ultrasound in Medicine, 600 Yishan Road, Shanghai, 200233 China

**Keywords:** Primary hyperparathyroidism, Papillary thyroid carcinoma, Ultrasound, Preoperative localization

## Abstract

**Background:**

The coexistence of primary hyperparathyroidism(PHPT) and papillary thyroid cancer(PTC) is a known entity; it is a rare and complicated setting for diagnostic imaging.

**Methods:**

After reviewing clinical data of 112 patients who had been treated for PHPT in our facility between January 2015 and December 2017, we identified 7 non-medullary thyroid carcinoma (NMTC) (6.25%). All of them had taken an ultrasound scan and undergone operation. In addition, we have also reviewed relevant reports from other facilities addressing PHPT and NMTC (Mainly PTC).

**Results:**

The 7 NMTCs were all pathologically confirmed PTC in our study, and they consisted of 6 parathyroid adenomas and 1 parathyroid carcinoma. 1 of the 7 patients had 2 malignant PTC nodules with neck lymph node metastasis, the rest 6 had single-focal PTC. Processing previous report data supported an association between PHPT and PTC, although the coexistence of PHPT and PTC is rare, but it does happen. Ultrasound, as an effective examination, would help screen the simultaneous lesions before operation, thus to avoid second surgery if not observed for both diseases at initial stage.

**Conclusions:**

Ultrasound is a necessary choice for preoperative localization, because it has the ability to simultaneously examine the thyroid and parathyroid lesions.

## Background

The coexistence of primary hyperparathyroidism (PHPT) and non-medullary thyroid carcinoma (NMTC) was initially described by Ogburn and Black in 1956. According to their reports, 3 cases of synchronous PHPT and NMTC (including PTC and FTC) of the thyroid glands in patients operated for parathyroid adenoma was found [1]. NMTC with PHPT has been reported in 2–11% of patients undergone surgery for PHPT [2]. The majority of the cases were uni-focal occult PTC without cervical lymph nodes involvement in women. Besides, many of the cases were associated with a previous head and neck irradiation [3]. The coexistence of PHPT and PTC is rare; and PHPT was usually considered as the primary pathology and was diagnosed before the identification of the thyroid carcinoma that was usually diagnosed in a pathology specimen as an incidental finding after parathyroid surgery. Such phenomenon would further complicate the management process, especially resulting in the need of a second surgery [2]; therefore, a carefully preoperative imaging would be necessary. We present 7 cases of synchronous PHPT and PTC, trying to explore the significance of ultrasound in preoperative localization; furthermore, relevant reports from other research centers addressing PHPT and NMTC (Mainly PTC) were also reviewed.

## Methods

We retrospectively studied 112 patients with PHPT admitted to our center between January 2015 and December 2017. Finally, 7 cases, pathologically confirmed as synchronous PHPT and PTC, were enrolled in. All the patients were healthy, and none of them had any risk factors related to thyroid cancer according to the American Thyroid Association (ATA) 2015 guidelines (i.e. prior thyroid cancer, family history, or exposure to external irradiation) [4]. And all of them had cervical ultrasound scan suggesting parathyroid lesion and were scheduled for an elective parathyroidectomy. The ultrasound scans were all operated by Professor Yan Wang, the corresponding author of this article. The number, size, location, border, blood supply and other relative detections were recorded. The ultrasonography was observed by using a linear probe of SIMENS 3000 and/or HITACH with a frequency of 8-12 MHz. Moreover, some patients were chosen to undergo 99mTc-MIBI or CT selectively. Surgery was achieved in patients who met the criteria for parathyroidectomy. As there were other thyroid lesions suspicious for PTC, the thyroid specimen was sent for an intraoperative frozen section pathologic examination, which confirmed PTC. Based on above information and consent approved, lobectomy or total thyroidectomy was performed in addition to the minimally invasive parathyroidectomy (MIP). The PUBMED and EMBASE electronic database was searched to identify relevant studies among recent years. With this purpose, we used the following terms: “primary hyperparathyroidism” and “non-medullary thyroid carcinoma” but limited to “human”. Moreover, relevant studies were also extensively searched by hand; language limitations were applied as English.

## Results

### Patients

A total of 112 patients who underwent parathyroidectomy for PHPT were selected. All the patients had taken a cervical ultrasound scan. Mean age of 7 patients with synchronous PHPT and PTC was 50 years (34–57 y) and 6/7 (85.7%) were female. None of these patients had a history of radiation exposure. 1 of the 7 patients underwent lobectomy, with the pathological diagnosis of thyroid adenoma. Preoperatively, the serum calcium of these patients was 2.51~3.63 mmol/L (Normal 2.09~2.6 mmol/L), serum phosphate was 0.75~1.34 mmol/L (Normal 0.8~1.6 mmol/L), and serum parathyroid hormone was 57.99~ 425 pg/mL (Normal 15~ 65 pg/mL).

In most case reports describing the coexistence of these two lesions, PHPT was usually diagnosed before the identification of the thyroid carcinoma which was usually diagnosed in pathology specimen as an incidental finding after the surgery [2, 5, 6]. However, in our study, most of the cases were admitted by annual thyroid nodule examination, and the parathyroid lesion was prompted by following ultrasound (Table 1).

**Table 1:** Patient characteristics and disease etiology

Characteristic	Patient							
	1	2		3	4	5	6	7
Gender	F	F		F	F	M	F	F
Age, years	56	34		57	54	52	50	49
Reason	Thyroid nodule follow-up.	Bone pain (mainly right upper limb) for half a year.		Thyroid nodule follow-up.	Thyroid nodule follow-up.	Thyroid and parathyroid nodule during annual examination	Thyroid nodule follow-up.	Thyroid nodule follow-up.
History	No history of radiation exposure	·Lobectomy history (Left lobe-thyroid adenoma).·Brown tumor.·No history of radiation exposure		No history of radiation exposure	No history of radiation exposure	No history of radiation exposure	No history of radiation exposure	No history of radiation exposure
Preoperative biochemistry								
Calcium, mg/dL	2.65	3.63		2.66	3.01	2.75	2.51	2.98
Phosphate, mg/dL	1.15	0.98		1.34	0.87	–	0.95	0.75
Parathyroid hormone, pg/mL	189.9	769.2		57.99	164.3	114.9	86.87	425
Ultrasound features								
Number	1	2		1	1	1	1	1
Echo	Hypoechoic/Homogeneous/Calcification	Hypoechoic/Homogeneous	Hypoechoic/Heterogeneous	Hypoechoic/Heterogeneous	Hypoechoic/Heterogeneous	Hypoechoic/Homogeneous	Hypoechoic/Homogeneous	Hypoechoic/Homogeneous/Calcification
Size	10 mm × 6 mm	12 mm× 8 mm	22 mm × 15 mm × 32 mm	21 mm × 10 mm× 5 mm	16 mm× 9 mm	23 mm × 9 mm	12 mm × 7 mm	7.5 mm × 7.2 mm
Location	Posterior to the right thyroid lobe, lower pole	Inferior pole of the right thyroid lobe, adjacent to the trachea	Zone of left thyroid lobe	Near the upper pole, posterior to the right thyroid lobe	Inferior pole of the left thyroid lobe	Posterior to the middle right thyroid lobe, adjacent to the trachea	Near the superior pole of the right thyroid lobe	Inferior pole of the left thyroid lobe
Form	regular	regular	irregular	regular	regular	regular	regular	irregular
Border	clear	unclear	unclear	clear	clear	clear	clear	clear
Blood	II	III	III	III	III	III	III	III
Elastography	1	2	2	–	–	–	–	3
Other	Hypoechoic thyroid nodule measuring 8.6 mm × 9.1 mm to the upper pole of left lobe with irregular form, unclear border, calcification, a little blood. Elastography grade: 4.	Several hypoechoic thyroid nodules and cysts in the right lobe.		Hypoechoic thyroid nodule measuring 5.9 mm× 6.8 mm in the middle of right lobe next to the anterior membrane, taller-than-wide, irregular form, unclear border. No blood detected.	Hypoechoic thyroid nodule measuring 14 mm× 6 mm in the left lobe with irregular form, unclear border, micro-calcification, a little blood.	Several small (Diameter 3 mm to 10 mm) hypoechoic thyroid nodules highly suspicious malignant according to the ATA 2015 guideline.	Hypoechoic thyroid nodule measuring 11 mm × 7 mm near the inferior pole of the left thyroid lobe with irregular form, unclear border, micro-calcification, a little blood. Elastography grade: 4. Other two nodules of very low suspicious.	Several hypoechoic and mixed echo thyroid nodules in both lobes. The biggest one in the right lobe was in size of 23 mm× 16 mm with irregular form, unclear border, arc-like calcification, a little blood.
99mTc-MIBI or CT features	–	Cervical CT: Right lobe nodules, left side lesion suspicious series from parathyroid		–	99mTc-MIBI: Highly suspicious of left side parathyroid adenoma.	–	99mTc-MIBI: Suspicious of left side PHPT.Cervical CT: Nodule in the left lobe of malignant suspicious. Nodule inferior to the right lobe can not excepted the possibility of parathyroid original.	Cervical CT: Multiple nodules in both thyroid lobes.

### Ultrasound

**Fig. 1:**
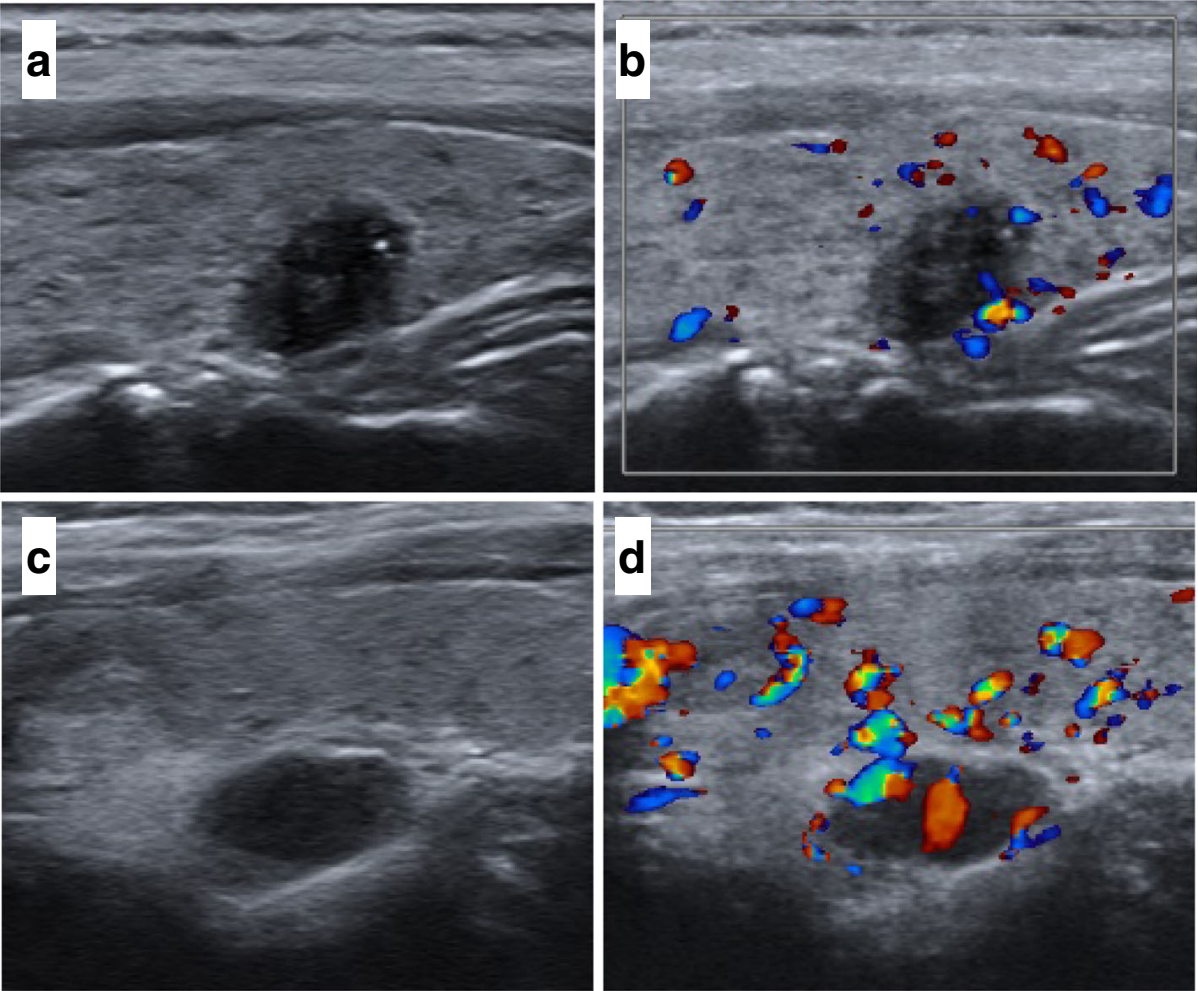
Cervical ultrasonography of a 50-year-old female patient. **a** and **b** showed a hypoechoic thyroid nodule measuring 11 mm× 7 mm near the inferior pole of the left thyroid lobe with irregular form, unclear border, micro-calcification. Little blood signal was detected in CDFI mode. **c** and **d** showed a hypoechoic right superior parathyroid lesion. Rich color blood flow signal was detected. The pathological results were papillary thyroid micro-carcinoma and parathyroid adenoma respectively

Among the 7 patients, totally 8 hypoechoic parathyroid nodules were observed by ultrasound, 5 of which were located on the right side of the patients’ necks. The result is similar in comparison with former studies [2]. The size of the nodules varied from the smallest one measuring 7.5 mm× 7.2 mm to the biggest measuring 32 mm× 22 mm× 15 mm. Among them, 2 of the 8 nodules, including 1 adenoma and 1 carcinoma, were observed to have calcification. 4 of the 7 patients were found to have multiple thyroid nodules including hypoechoic nodules, mixed-echo nodules and cysts; and at least one of them was considered malignant according to the guideline (Table 1, Fig. 1).

### CT and ^99^Tc^m^-MIBI SPECT

We have implemented ^99^Tc^m^-MIBI on 3 patients; the results showed suspicious or highly suspicious of parathyroid lesion. 3 patients underwent cervical CT, 2 of which prompted suspicious parathyroid lesions (Table 1). Moreover, by comparison of 3 imaging examinations, ultrasound is more efficient than cervical CT and ^99^Tc^m^-MIBI (Table 2).

**Table 2:** Comparisons of US, CT and 99mTc-MIBI

Patient		1		2		3		4		5		6		7	
Characteristics		Th	Pth	Th	Pth	Th	Pth	Th	Pth	Th	Pth	Th	Pth	Th	Pth
Finding^a^	US	√	√	√	√	√	√	√	√	√	√	√	√	√	√
	CT	–	–	√	√	–	–	–	–	–	–	√	√	√	×
	MIBI	–	–	–	–	–	–	×	√	–	–	×	√	–	–
Diagnosis^a^	US	√	√	√	√	√	√	√	√	√	√	√	√	√	×
	CT	–	–	√	Suspicious^b^	–	–	–	–	–	–	√	Cannot except the possibility^c^	√	×
	MIBI	–	–	–	–	–	–	×	√	–	–	×	Suspicious	–	–

*Abbreviations*: *Th* Thyroid lesion, *Pth* Parathyroid lesion. ^a^ Finding: The lesion could be detected during or after the imaging scan; Diagnosis: The origin of the lesions (thyroid or parathyroid) can be correctly identified according to the imaging. ^b^Suspicious: About 60% of the Possibilities. ^c^Cannot except the possibility: Lower than 50% of the Possibilities

### Pathological results and surgical managements

Surgery was achieved in patients who met the criteria for parathyroidectomy; as there were other thyroid lesions suspicious for PTC, the thyroid specimen was sent for intraoperative frozen section pathologic examinations, which confirmed PTC. According to the intraoperative frozen results, which revealed all the suspected thyroid nodules in ultrasound were malignant; 2 patients underwent total thyroidectomies and 5 received lobectomies. Moreover, necessary exploration of recurrent laryngeal nerve and dissection of central cervical lymph nodes were also performed. The final pathological results revealed that the cause of PHPT was a single parathyroid adenomatous gland in 5 (71.4%) patients, two parathyroid adenomatous glands in 1 (14.3%) patient, and parathyroid carcinoma in 1 (14.3%) patient. Besides, the final pathology of the thyroid nodules was PTMC in 4 patients and PTC in other 3 patients, and 1 of the 7 patients was multi-focal PTC with metastasis of four cervical central lymph nodes (Table 3).

**Table 3:** Intraoperative course and pathological results

Characteristics		Patient						
		1	2	3	4	5	6	7
Intraoperative findings		Lesion A1 in the left thyroid lobe, toughLesion A2 (about 1 cm) posterior to the right thyroid lobe, soft	Lesion B1 (2x3cm) in the left thyroid zone, adherence to adjacent tissueLesion B2 inferior pole of the right thyroid lobeLesion B3 in the right thyroid lobe	Lesion C1 in the right thyroid lobe, toughLesion C2 posterior to the right thyroid lobe	Lesion D1 in the left thyroid lobe, toughLesion D2 (about 1 cm) posterior to the left thyroid lobe, soft	Several nodules in both thyroid lobe, toughMultiple enlarged lymph nodes in the cervical central regionLesion E posterior to the right thyroid lobe	Lesion F1 in the left thyroid lobe, toughLesion F2 (about 1 cm) posterior to the left thyroid lobe	Lesion G1 (2.5 cm) in the right thyroid lobe, toughLesion G2 (4 cm) in the left thyroid lobe
Section frozen results		Lesion A1: PTCLesion A2: Parathyroid adenoma	Lesion B1: Wait for final pathologic resultLesion B2: Parathyroid adenomaLesion B3: PTMC (about 0.3 cm)	Lesion C1: PTC (0.4 cm)Lesion C2: Highly suspected parathyroid adenoma, wait for final pathologic result	Lesion D1: PTC (1.3 × 1 × 0.6 cm)Lesion D2: Parathyroid adenoma	Lesion E: Parathyroid adenomaBoth thyroid lobe: Multi-focal PTC (3 in the left, 1 in the right)	Lesion F1: PTC(0.5 cm)Lesion F2: Parathyroid adenoma	Lesion G1: PTC(2 × 1.5x1cm)Lesion G2: Thyroid adenoma with cystic degeneration and interstitial fibrosis; Suspected parathyroid carcinoma, wait for final pathologic result
Surgical approach		Left thyroid lobectomy + Dissection of left central cervical lymph nodes + Exploration of left recurrent laryngeal nerve + Right parathyroidectomy	Bilateral parathyroidectomy+ Right thyroid lobectomy + Dissection of right central cervical lymph nodes + Exploration of bilateral recurrent laryngeal nerve	Right thyroid lobectomy + Dissection of right central cervical lymph nodes + Exploration of right recurrent laryngeal nerve + Right parathyroidectomy	Left thyroid lobectomy + Dissection of left central cervical lymph nodes + Exploration of left recurrent laryngeal nerve + Left parathyroidectomy	Total thyroidectomy + Dissection of central cervical lymph nodes + Right parathyroidectomy	Left thyroid lobectomy + Dissection of left central cervical lymph nodes + Exploration of left recurrent laryngeal nerve + Left parathyroidectomy	Total thyroidectomy + Exploration of bilateral recurrent laryngeal nerve + Dissection of right central cervical lymph nodes
Pathological results	Parathyroid	Parathyroid adenoma	Parathyroid adenoma (Lesion B1 & B3)	Parathyroid adenoma	Parathyroid adenoma	Parathyroid adenoma	Parathyroid adenoma	Parathyroid carcinoma
	Thyroid	PTMC	PTMC	PTMC	PTC	Multi-focal PTC with metastasis of cervical lymph nodes	PTMC	PTC and Thyroid adenoma
Consistency with US		Y	Y	Y	Y	Y	Y	Y/N*

*Abbreviation*: *Y* Consistent, *N* Inconsistent. * The results of US report did not mention parathyroid lesion, but it was described in the image features. So it can’t be considered simply to be consistent or inconsistent

## Discussion

The coexistence of primary hyperparathyroidism and papillary thyroid cancer is a known entity over the years. From the initial report by Ogburn and Black in 1960s to the recent discussions over these years, a series of relevant clinical studies were conducted. According to their findings, it has been reported with a rate of 2~11% patients underwent surgery for PHPT [2]. As for the data we summarized, the average rate is about 3.5% of thyroid cancer among patients with PHPT undergoing parathyroidectomy (ranging 1.7 to 15%, 313 thyroid carcinoma cases among 9051 patients with PHPT) (Table 4). The largest cohort was described by Linos et al., who found 2.5% (51 of 2058) patients with surgically proved PHPT had associated NMTC [7]. Furthermore, the incidence rate of concurrence of PTC and PHPT in 5 big clinical series (the total number of patients with PHPT was over 500) is relatively stable of 2.1 to 3.3% [2, 8–10]. However, it was of great fluctuations in the small clinical series especially when the total number of patients being reviewed were less than 100 [11–13] (Table 4). The reason we analyzed from a statistic perspective is the high false positive rate caused by selection bias of small sample which also occurred in our data (6.25%). Combining with statistic experience, the data from relevant big sample is more convincing due to the low incidence of synchronous PHPT and PTC. On the other hand, the synchronous PTC and PHPT has a concurrence rate between 2.6 to 4.5% in the patients firstly admitted for thyroid operation, and Niedźwiecki et al. considered that there was no significant difference of PHPT incidence between various type of goiter [14].

**Table 4:** Papillary thyroid cancer among patients with PHPT undergoing parathyroidectomy

Author	Year	Number of patients with PHPT	Number of patients with NMTC	Incident rate(%)
Linos et al. [4]	1982	2058	51	2.5
Lehwald et al. [2]	2013	1464	41	2.8
Attie and Vardhan [8]	1993	948	31	3.3
Burmeister et al. [9]	1997	700	18	2.6
Bentrem et al. [10]	2002	580	12	2.1
Hedman and Tisell [21]	1984	426	25	5.8
Nishiyama et al. [22]	1979	420	13	3.1
Strichartz and Giuliano [23]	1990	388	11	2.8
Prinz et al. [24]	1982	351	16	4.6
Krause et al. [25]	1996	322	9	2.8
LiVolsi et al. [26]	1976	272	31	11.4
Ogburn and Black [1]	1956	230	4	1.7
Phillips et al. [27]	2014	217	5	2.3
Morita et al. [28]	2008	200	12	6.0
Xue et al. [29]	2016	155	12	7.7
Beus et al. [30]	2004	101	3	3.0
Arciero et al. [11]	2012	94	6	6.4
Sidhu and Campbell [12]	2000	65	4	6.2
Gul et al. [13]	2010	60	9	15.0
Total	–	9051	313	3.5

The mechanisms underlying the relationship between PHPT and PTC have still left to be unknown; most of the published studies claimed this relationship is still considered as coincidental. Previous head and neck irradiation in childhood appears to be an increased risk for the development of both PHPT and NMTC [3, 15–17]. However, many patients included in the previous clinical series did not have a radiation exposure or treatments; neither did we found in the patients in our study. Thus, it may not be an essential risk for the concurrence of PHPT and PTC. Beebeejaun et al. suggested a possible hypothesis for the correlation based on shared embryological origin and genes (i.e. Eya 1), high parathyroid hormone (PTH), low 1,25 hydroxyl vitamin D, hypercalcemia resulting in high levels of angiogenic growth factors (i.e. bFGF) [18]. Hypotheses have been presented, but no firm conclusions exist at this time regarding the etiology of synchronous thyroid and parathyroid disease.

Over the past years, more and more articles related to coexistence of PHPT and NMTC were reported, but the specific treatment strategy in guidelines has not been established. For PHPT, modern surgical management has transitioned from the traditional bilateral neck exploration to minimally invasive parathyroidectomy. This surgical approach allows for smaller incisions, lower morbidity, but less exposure of the thyroid glands, which leads to concerns about missing coexistent thyroid pathology. As for NMTC, total thyroidectomy or lobectomy with necessary cervical lymph nodes dissection is consensus according to the American Thyroid Association (ATA) guideline. Based on above, minimally invasive parathyroidectomy for PHPT with selective total thyroidectomy or lobectomy for thyroid nodules seems to be the main way currently.

Properly surgical approach depends on accurate preoperative imaging, several imaging procedures have been described for the preoperative localization of parathyroid tumors in the present era of minimally invasive parathyroidectomy. Among these methods, ^99^Tc^m^-sestamibi is usually recommended as the first choice [19], while ultrasound seems just as a supplement without enough attention. Reviewing previous studies listed in Table 3, it has barely mentioned the role of ultrasound before operation; however, as an inexpensive and noninvasive technique, ultrasound acts as the perfect diagnostic tool to detect concomitant thyroid and parathyroid nodules. For the patients ready for the operation of PHPT, as ultrasound screening was routinely performed, thyroid nodules are not easy to missed diagnosis [11]. Here comes another question: If the patient is admitted for NMTC for the first time as the patients in our study, what we could do to avoid missing the parathyroid lesion as much as possible?

In most previous reports like Lehwald et al., PHPT was usually diagnosed before the identification of the thyroid carcinoma that was usually diagnosed in pathology specimen as an incidental finding after the surgery [2]; however, in our study, most of the cases were admitted by annual thyroid nodule examination, while the parathyroid lesion was not prompted until taking an ultrasound screening before the surgery. In this current study, 6 of 7 patients were discovered of suspected parathyroid lesion as described in the ultrasound reports, of which 5 patients did not perform serum PTH test before ultrasound. The following high level of serum PTH basically certified our suspicion before the operation. Among the patients, only 1 of them had the main complaint of bone pain, and her serum PTH level was in normal during two tests before surgery. One interesting case is one of the patients was not prompted parathyroid lesion in the ultrasound results; but this patient was asymptomatic hyperparathyroidism and there was no evidence supporting abnormal serum PTH level before ultrasound. By summarizing the clinical data of this female patient and comparing intraoperative findings and ultrasound reports, we found the lesion in the same position was misdiagnosed as PTC without the reminder of high level of PTH (Tables 1 and 3). Furthermore, Nam et al., who had analyzed 7 parathyroid cancers and 32 parathyroid adenomas, noted that the significant ultrasound features of parathyroid carcinoma includes: large size, heterogeneous echotexture, irregular shape, non-circumscribed margin, intra-nodular calcifications, and local invasion [20]. All of the imaging features of parathyroid carcinomas are similar to those of PTCs’. Although accurate diagnosis was not given, we succeeded to find out the malignant lesion at least. We thought more accurate diagnosis would have arrived if the PTH level was in unusual level before the ultrasound exam.

## Conclusion

In conclusion, our study illustrates the need for clinical awareness of concomitant hyperparathyroidism and non-medullary thyroid cancer and is substantiated with published case reviews. The coexistence of PHPT and NMTC is rare but it does happen. This study, together with other findings, concluded that there is some relationship during the concurrence procedure of PHPT and PTC; moreover, comprehensive preoperative ultrasound of both thyroid and parathyroid glands is necessary for patients with PHPT. To date, there is still no specific guidelines for the management of patients with synchronous PHPT and PTC, so the treatment that which one of the two is dominant to deal with is still controversial; early detection maybe can do something to promote the process of the management coming to a consensus.

Different from previous opinions that considering ^99^Tc^m^-sestamibi as the first choice, we think that ultrasound seems a more efficient and necessary option for preoperative localization, for it can simultaneously screen the thyroid and parathyroid lesion. If combined with biochemical tests, the rate of missing diagnosis or misdiagnosis will be sharply reduced. All of these will help to contribute for a precise surgery.

## Abbreviations

NTMC: Non-medullary thyroid carcinoma
PHPT: Primary hyperparathyroidism
PTC: Papillary thyroid carcinoma

## References

[1] Fau OP, Black BM (1956). Primary hyperparathyroidism and papillary adenocarcinoma of the thyroid; report of four cases.

[2] Lehwald N, Cupisti K, Krausch M, Ahrazoglu M, Raffel A, Knoefel WT (2013). Coincidence of primary hyperparathyroidism and nonmedullary thyroid carcinoma. Horm Metab Res.

[3] Wilson SD, Doffek KM, Wang TS, Krzywda EA, Evans DB, Yen TW (2011). Primary hyperparathyroidism with a history of head and neck irradiation: the consequences of associated thyroid tumors. Surgery.

[4] Haugen BR, Alexander EK, Bible KC, Doherty GM, Mandel SJ, Nikiforov YE (2015). American Thyroid Association management guidelines for adult patients with thyroid nodules and differentiated thyroid Cancer: the American Thyroid Association guidelines task force on thyroid nodules and differentiated thyroid Cancer. Thyroid.

[5] Aşik M, Anaforoǧlu I, Köse M, Karyagar S, Mollamehmetoǧlu B, Algün E (2013). Papillary thyroid carcinoma with primary hyperparathyroidism: a report of two cases and a brief literature review. Turkish J Endocrinol Metab.

[6] Polyzos SA, Anastasilakis AD, Iakovou IP, Partsalidou V (2010). Primary hyperparathyroidism and incidental multifocal metastatic papillary thyroid carcinoma in a man. Arq Bras Endocrinol Metabol.

[7] Linos DA, van Heerden JA, Edis AJ (1982). Primary hyperparathyroidism and nonmedullary thyroid cancer. Am J Surg.

[8] Attie JN, Vardhan R (1993). Association of hyperparathyroidism with nonmedullary thyroid carcinoma: review of 31 cases. Head Neck..

[9] Burmeister LA, Sandberg M, Carty SE, Watson CG (1997). Thyroid carcinoma found at parathyroidectomy: association with primary, secondary, and tertiary hyperparathyroidism. Cancer.

[10] Bentrem DJ, Angelos P, Talamonti MS, Nayar R (2002). Is preoperative investigation of the thyroid justified in patients undergoing parathyroidectomy for hyperparathyroidism?. Thyroid.

[11] Arciero CA, Shiue ZS, Gates JD, Peoples GE, Dackiw AP, Tufano RP (2012). Preoperative thyroid ultrasound is indicated in patients undergoing parathyroidectomy for primary hyperparathyroidism. J Cancer.

[12] Sidhu S, Campbell P (2000). Thyroid pathology associated with primary hyperparathyroidism. Aust N Z J Surg.

[13] Gul K, Ozdemir D, Korukluoglu B, Ersoy PE, Aydin R, Ugras SN (2010). Preoperative and postoperative evaluation of thyroid disease in patients undergoing surgical treatment of primary hyperparathyroidism. Endocr Pract.

[14] Niedźwiecki S, Kuzdak K, Kaczka K, Pomorski L (2007). Normocalcemic, subclinical, asymptomatic primary hyperparathyroidism in patients with goiter or papillary thyroid cancer--preliminary report. Normocalcemic primary hyperparathyroidism and thyroid pathology. Wiadomości lekarskie (Warsaw, Poland : 1960).

[15] Cohen J, Gierlowski TC, Schneider AB (1990). A prospective study of hyperparathyroidism in individuals exposed to radiation in childhood. JAMA.

[16] Stephen AE, Chen KT, Milas M, Siperstein AE (2004). The coming of age of radiation-induced hyperparathyroidism: evolving patterns of thyroid and parathyroid disease after head and neck irradiation. Surgery.

[17] Woll M, Sippel RS, Chen H (2011). Does previous head and neck irradiation increase the chance of multigland disease in patients with hyperparathyroidism?. Ann Surg Oncol.

[18] Beebeejaun M, Chinnasamy E, Wilson P, Sharma A, Beharry N, Bano G (2017). Papillary carcinoma of the thyroid in patients with primary hyperparathyroidism: is there a link?. Med Hypotheses.

[19] Leitha T, Staudenherz A (2003). Concomitant hyperparathyroidism and nonmedullary thyroid cancer, with a review of the literature. Clin Nucl Med.

[20] Nam M, Jeong HS, Shin JH (2017). Differentiation of parathyroid carcinoma and adenoma by preoperative ultrasonography. Acta Radiol.

[21] Hedman I, Tisell LE (1984). Associated hyperparathyroidism and nonmedullary thyroid carcinoma: the etiologic role of radiation. Surgery.

[22] Nishiyama RH, Farhi D, Thompson NW (1979). Radiation exposure and the simultaneous occurrence of primary hyperparathyroidism and thyroid nodules. Surg Clin N Am.

[23] Strichartz SD, Giuliano AE (1990). The operative management of coexisting thyroid and parathyroid disease. Arch Surg.

[24] Prinz RA, Barbato AL, Braithwaite SS, Brooks MH, Emanuele MA, Gordon DL (1982). Simultaneous primary hyperparathyroidism and nodular thyroid disease. Surgery.

[25] Krause UC, Friedrich JH, Olbricht T, Metz K (1996). Association of primary hyperparathyroidism and non-medullary thyroid cancer. Eur J Surg.

[26] LiVolsi VA, Feind CR (1976). Parathyroid adenoma and nonmedullary thyroid carcinoma. Cancer.

[27] Phillips DJ, Kutler DI, Kuhel WI (2014). Incidental thyroid nodules in patients with primary hyperparathyroidism. Head Neck.

[28] Morita SY, Somervell H, Umbricht CB, Dackiw AP, Zeiger MA (2008). Evaluation for concomitant thyroid nodules and primary hyperparathyroidism in patients undergoing parathyroidectomy or thyroidectomy. Surgery.

[29] Xue Y, Ye ZQ, Zhou HW, Shi BM, Yi XH, Zhang KQ (2016). Serum calcium and risk of nonmedullary thyroid Cancer in patients with primary hyperparathyroidism. Med Sci Monit.

[30] Beus KS, Stack BC (2004). Synchronous thyroid pathology in patients presenting with primary hyperparathyroidism. Am J Otolaryngol.

